# Anlotinib Enhances the Antitumor Activity of High-Dose Irradiation Combined with Anti-PD-L1 by Potentiating the Tumor Immune Microenvironment in Murine Lung Cancer

**DOI:** 10.1155/2022/5479491

**Published:** 2022-02-01

**Authors:** Meng Yuan, Yirui Zhai, Yu Men, Maoyuan Zhao, Xin Sun, Zeliang Ma, Yongxing Bao, Xu Yang, Shuang Sun, Yunsong Liu, Wanting Zhang, Zhouguang Hui

**Affiliations:** ^1^Department of Radiation Oncology, National Cancer Center/National Clinical Research Center for Cancer/Cancer Hospital, Chinese Academy of Medical Sciences and Peking Union Medical College, Beijing, China; ^2^Department of VIP Medical Services, National Cancer Center/National Clinical Research Center for Cancer/Cancer Hospital, Chinese Academy of Medical Sciences and Peking Union Medical College, Beijing, China; ^3^Lung Cancer Center, West China Hospital of Sichuan University, Chengdu, Sichuan, China; ^4^Department of Radiation Oncology, The First Affiliated Hospital, Zhejiang University School of Medicine, Hangzhou, Zhejiang, China

## Abstract

**Background:**

Radioimmunotherapy has become one of the most promising strategies for cancer treatment. Preclinical and clinical studies have demonstrated that antiangiogenic therapy can improve the efficacy of immunotherapy and sensitize radiotherapy through a variety of mechanisms. However, it is undefined whether angiogenesis inhibitors can enhance the effect of radioimmunotherapy. In this study, we aim to explore the role of anlotinib (AL3818) on the combination of radiotherapy and immune checkpoint inhibitors in Lewis lung carcinoma mouse.

**Methods:**

C57BL/6 mouse subcutaneous tumor model was used to evaluate the ability of different treatment regimens in tumor growth control. Immune response and immunophenotyping including the quantification and activation were determined by flow cytometry, multiplex immunofluorescence, and multiplex immunoassay.

**Results:**

Triple therapy (radiotherapy combined with anti-PD-L1 and anlotinib) increased tumor-infiltrating lymphocytes and reversed the immunosuppressive effect of radiation on the tumor microenvironment in mouse model. Compared with radioimmunotherapy, the addition of anlotinib also boosted the infiltration of CD8^+^ T cells and M1 cells and caused a decrease in the number of MDSCs and M2 cells in mice. The levels of IFN-gamma and IL-18 were the highest in the triple therapy group, while the levels of IL-23, IL-13, IL-1 beta, IL-2, IL-6, IL-10, and Arg-1 were significantly reduced. NF-*κ*B, MAPK, and AKT pathways were downregulated in triple therapy compared with radioimmunotherapy. Thus, the tumor immune microenvironment was significantly improved. As a consequence, triple therapy displayed greater benefit in antitumor efficacy.

**Conclusion:**

Our findings indicate that anlotinib might be a potential synergistic treatment for radioimmunotherapy to achieve better antitumor efficacy in NSCLC patients by potentiating the tumor immune microenvironment.

## 1. Introduction

Lung cancer remains the most commonly diagnosed cancer and the leading cause of cancer-related death worldwide [1]. Radiotherapy (RT) is an indispensable local treatment method in the treatment of inoperable lung cancer [2]. Despite the success of radiotherapy, it cannot always eradicate the primary tumor, and local recurrence and metastasis are still observed following irradiation, indicating the inadequacy of RT-induced response to maintain antitumor immunity. Immunotherapy, especially the immune checkpoint inhibitor (ICI), has become the most active part in the research of cancer treatment in the past decade [3–5]. Although immunotherapy can produce even more consistent clinical benefits than chemotherapy or targeted therapy, only about 20% patients would respond to immunotherapy alone [6, 7]. RT, particularly stereotactic body radiation therapy (SBRT) also known as stereotactic ablative radiotherapy (SABR), can promote the antitumor immunity through induction of an immunogenic cell death and in situ vaccination in preclinical studies [8, 9]. Recently, with a robust and growing body of clinical data, RT has been demonstrated to augment the antitumor immune responses elicited by immunotherapy through synergistic effects [10–12]. The combination of RT and ICI has become one of the most promising strategies for cancer treatment.

Even though the brilliant future of RT-ICI combination therapy, radiation, or drug resistance may occur in clinic. How to optimize it has become a hotspot, including how to further improve the response rate and scale up the potential population who can benefit and reduce the incidence rate of adverse events related to combination treatment. Antiangiogenic therapy, represented by a class of vascular endothelial growth factor (VEGF) inhibitors, is an important component of targeted therapy [13]. There is growing evidence that antiangiogenic therapy can improve both radiotherapy and immunotherapy by downregulating the level of angiogenic factors, normalizing blood vessels, alleviating hypoxia, adjusting cell cycle, balancing the tumor microenvironment, and eliminating immunosuppressive factors to some degree [14–16]. However, the evidence of the trinity treatment combining radiotherapy, immunotherapy, and antiangiogenic therapy is rare. In the light of the previously described interactions and synergy, it is becoming increasingly important to explore whether antiangiogenic therapy could enhance the therapeutic effect of radiotherapy combined with immunotherapy, and the trinity strategy could bring about even dramatically tumor shrinking. Therefore, the aim of our study is to comprehensively characterize the role of antiangiogenic therapy on the treatment efficacy of radioimmunotherapy and the tumor microenvironment. Our data suggested a novel possible therapeutic approach for cancer patients, and this treatment paradigm merits evaluation in clinical trials in the future.

## 2. Methods

### 2.1. Mice and Cell Lines

Female C57BL/6 mice (SPF, 6 weeks old) were purchased from HFK Bioscience Co., Ltd. (Beijing, China). All mice were group-housed (5 mice/cage) in the Experimental Animal Center of the Chinese Academy of Medical Sciences and were in specific pathogen-free conditions under a 12 h light-dark schedule. The temperature and humidity of the animal house were maintained at 26°C–28°C and 60 ± 5%, respectively. In vivo study protocols were approved by the Institutional Animal Care and Use Committee (NCC2020A305) at the National Cancer Center/Cancer Hospital, Chinese Academy of Medical Sciences (NCC/CH, CAMS, Beijing, China). Lewis lung carcinoma cells (LLC) were kindly provided by Dr. Li at CAMS Key Lab of Translational Research on Lung Cancer and were cultured in complete DMEM supplemented with 10% FBS, 100 U/ml penicillin, and 100 *μ*g/ml streptomycin at 37°C in a 5% CO2 atmosphere.

### 2.2. Establishment of Subcutaneous Tumor Models

To establish a mouse model with normal immune system, C57BL/6 mice were subcutaneously implanted with LLC cells (6 × 10^6^) in the left inguinal region. Tumor volumes were measured every other day using a caliper and calculated by the following formula: volume = *L* × *W*^2^/2, where *L* was the length and *W* the width of the tumor. When tumor volume reached 50 ± 20 mm^3^, 10 days after implantation, mice were randomized into six groups of five animals each. The total rates of tumor formation of the subcutaneously implanted tumor cells were 75%. Then, the antitumor treatments began.

### 2.3. Antitumor Treatments

**Figure 1:**
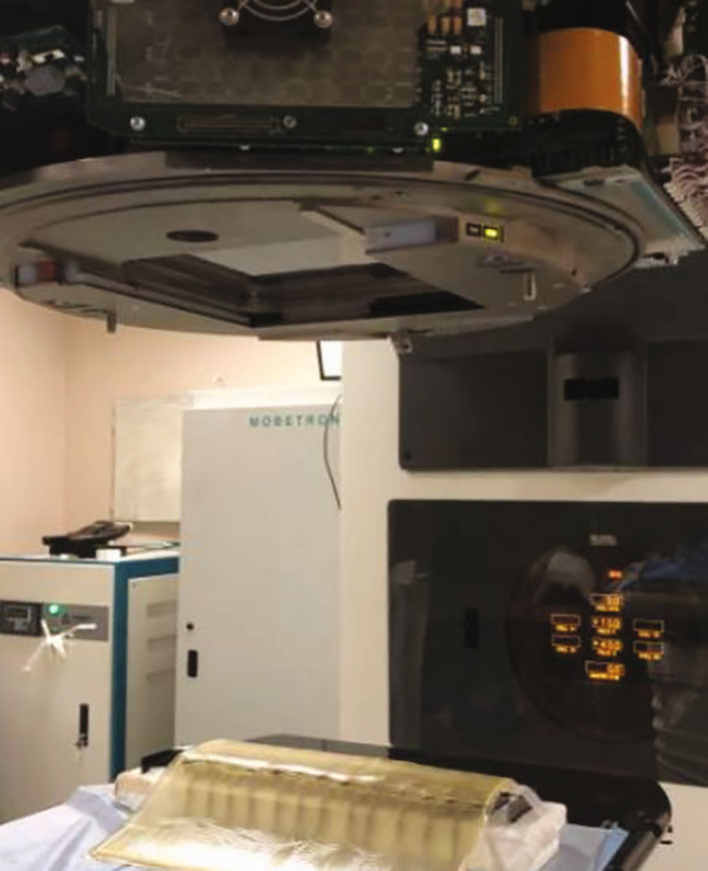
Radiation process of the tumor-burdened mice.

Mice were randomized into the following treatment groups: control, RT (24 Gy/3 f on days 3, 5, and 7), anti-PD-L1 (10 mg/kg, on days 3, 6, 9, and 12, clone 10F.9G2, BioXCell), RT+anlotinib (3 mg/kg, from day 1 to 14, #17321004, Chiatai Tianqing), RT+anti-PD-L1, and RT+anti-PD-L1+anlotinib. Mice received anti-PD-L1 by intraperitoneal injections and anlotinib by intragastric administration. RT (three fractions of 8 Gy) was delivered using a Varian Unique-SN2242 unit at 300 cGy per minute (100 cm source to skin distance). Each mouse was anesthetized with chloral hydrate (30 mg/kg), placed supinely on a fixing device, covered with a 5 mm bolus, and left only the tumor area exposed (Figure 1). Endpoint day was designated as the 15th day after commencement of the initial treatment to harvest tumors. For ethical considerations, mice with a weight loss of 20% or more, tumor diameter larger than 20 mm, or tumor causing severely impaired ambulation were euthanized.

### 2.4. Flow Cytometry

The harvested tumor tissues were digested with 1 mg/ml collagenase IV (Sigma-Aldrich) and 0.01 mg/ml DNAase (Sigma-Aldrich) at 37°C for 60 minutes. Cell suspensions were passed through a 70 *μ*m nylon cell strainer to yield single-cell suspensions. The resulting single-cell suspensions were stained with fluorescent-labeled antibodies, including anti-CD3 (#100329, Biolegend), anti-CD4 (#100405, Biolegend), anti-CD8 (#100733, Biolegend), anti-CD45 (#103113, Biolegend), anti-PD-1 (#135217, Biolegend), anti-PD-L1 (#124311, Biolegend), anti-CD11b (#101245, Biolegend), anti-Gr-1 (#108407, Biolegend), anti-F4/80 (#123107, Biolegend), anti-CD86 (#105029, Biolegend), and anti-CD206 (#141707, Biolegend). The specific anti-mouse CD16/CD32 purified antibody (#70-0161, Tonbo Biosciences) was used to block the FC receptor and thus eliminate nonspecific results caused by binding of fluorescently labeled antibodies to Fc receptors. Isotype controls were used for all antibodies. Gates and quadrants were set based on isotype control staining. Data were acquired using an LSR-II (Becton Dickinson) and were analyzed using FlowJo V.10 software.

### 2.5. Immunofluorescence (IF)

Harvested tumors were fixed in 10% neutral-buffered formalin and embedded in paraffin. The tumor tissue sections (4 *μ*m) were then deparaffinized, rehydrated, and subjected to heat-induced antigen retrieval with Tris-EDTA buffer (pH 8.0). Sections were incubated in 10% normal goat serum at room temperature for 0.5 h. Tumor sections were then stained with primary antibodies (CD4, #GB11064, Servicebio; CD8, #GB11068, Servicebio; CD86, #GB13585, Servicebio and CD206, #GB13438, Servicebio) at 4°C overnight and corresponding secondary antibody marked with HRP for 50 minutes and then incubated with TSA-FITC (TSA-CY3) solution. Nuclei were stained with DAPI solution. Tumor tissue sections were observed with fluorescence microscopy.

### 2.6. Quantification of Cytokines in Tumor Tissues

Immune cytokine profiles were characterized using the ProcartaPlex Mouse Cytokine Panel (eBioscience). Tumor tissue samples were obtained from mice and run in three times along with serial standards and buffer controls. The median fluorescence intensity of analytes was detected using the Luminex 200. Cytokine concentrations were calculated with Luminex xPONENT V. 4.2 software using a standard curve derived from known reference concentrations supplied by the manufacturer. A five-parameter model was used to calculate final concentrations by interpolation.

### 2.7. Protein Extraction and Western Blot

Total protein was prepared from isolated tumor tissue, and Western blots were performed as previously described [17]. Primary antibodies include anti-MAPK (#4695, CST), anti-p-MAPK (#4370, CST), anti-AKT (#4691, CST), anti-p-AKT (#4060, CST), anti-NF-*κ*B (#8242, CST), anti-p-NF-*κ*B (#3033, CST), anti-arginase-1 (Arg-1) (#93668, CST), and anti-GAPDH (#5174, CST). Secondary antibodies include anti-rabbit (#14708, CST) and anti-mouse (#14709, CST).

### 2.8. Statistical Analysis

The data are shown as the means ± SEM and were analyzed using two-tailed Student's *T*-test for comparisons between two groups. One-way analysis of variance was used for the statistical analysis of more than two groups. ^∗^, ^∗∗^, ^∗∗∗^, or ^∗∗∗∗^ represents *p* values <0.05, <0.01, <0.001 or <0.0001, respectively. Statistical analyses were performed by the GraphPad Prism software 9.0 (GraphPad Software Inc., San Diego, CA, USA).

## 3. Results

### 3.1. Anlotinib Enhanced the Therapeutic Effect of Radiotherapy Combined with Immunotherapy, and the Triple Therapy Achieved the Best Antitumor Activity

**Figure 2:**
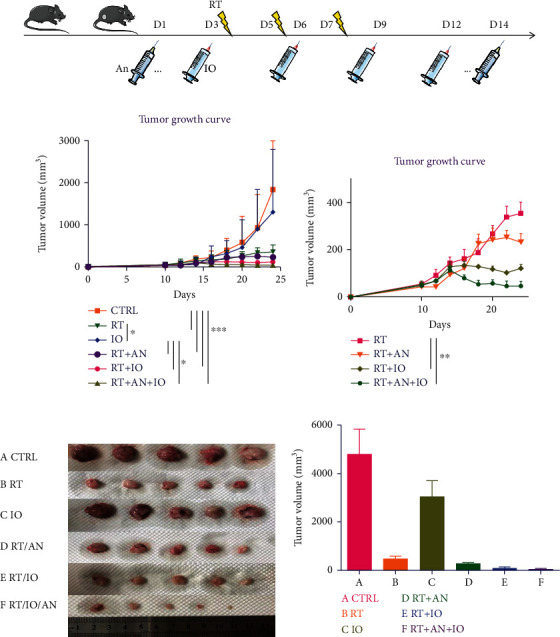
Radiotherapy combined with anti-PD-L1 and anlotinib achieved the best antitumor activity. (a) Schematic showing schedules of radiotherapy, anlotinib, and anti-PD-L1. (b–e) Responses of the subcutaneous tumors in different treatment groups. CTRL: control; RT: radiotherapy; IO: immunotherapy; AN: anlotinib.

Radiotherapy is a fundamental treatment for NSCLC, and radiotherapy combined with immunotherapy has been emerging as the most promising strategy, but there is no conclusion whether adding antiangiogenic therapy to this combination could exhibit even superior antitumor effect. To investigate the efficacy of triple therapy and the impact on the tumor immune microenvironment, we established the LLC mouse model with normal immune system. Considering the tolerance of multiple treatments and the accuracy of measurement, C57BL/6 mice were subcutaneously implanted with LLC cells (6 × 10^6^) in the left inguinal region. LLC cells were allowed to grow, allowing the tumors to become established 10 days after implantation (mean starting volume = 50 mm^3^). In the experimental groups, mice received one of the following treatments: RT, anti-PD-L1, RT+anlotinib, RT+anti-PD-L1, or RT+anti-PD-L1+anlotinib (Figure 2(a)). Hypofractionated radiotherapy was demonstrated to have brilliant synergetic effect with immunotherapy, and therefore, the irradiation was given as three fractions of 8 Gy and 24 Gy in total. We performed administration of anlotinib 2 days before RT considering of priming with the antiangiogenic therapy to produce a “treatment window” (Figure 2(a)). Anti-PD-L1 injections were given concurrently with and following radiotherapy to make a better synergy (Figure 2(a)). Tumor tissues (five mice each group) were collected on day 15 after the initial treatment. The tumor volumes of 6 groups were shown (Figures 2(b)–2(e)). To better replicate the clinical scenario, we treated LLC-bearing mice with various regimens, observing that compared with radiation alone, radioimmunotherapy and the triple therapy both significantly inhibited tumor growth. Triple therapy exhibited better antitumor efficacy than radioimmunotherapy (mean tumor volume ± SEM: RT: 487.5 ± 94.39 mm^3^ vs. radioimmunotherapy: 91.01 ± 32.19 mm^3^ vs. triple therapy: 46.33 ± 18.32 mm^3^; Figures 2(b)–2(e)).

What is more, the addition of anlotinib was well tolerated without weight decrease compared with other combination treatments. These data suggested that the triple therapy achieved the best antitumor activity and tolerable in mice.

### 3.2. Immunosuppressive Tumor Microenvironment following RT

**Figure 3:**
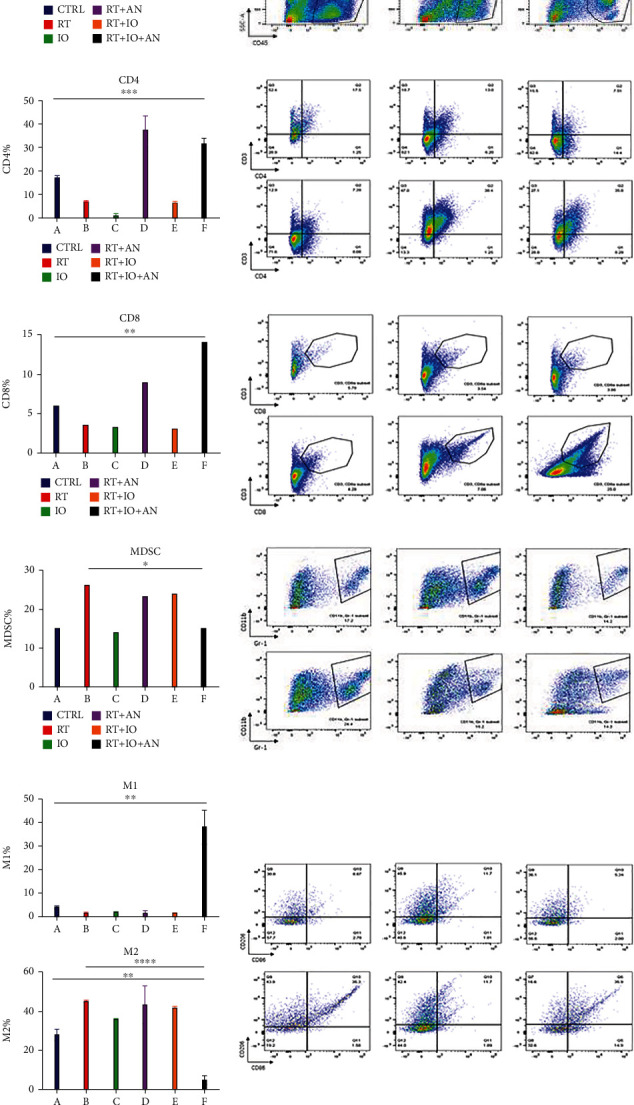
Radiotherapy combined with anti-PD-L1 and anlotinib could significantly potentiate the tumor immune microenvironment compared with other treatment combinations. Quantitative data and flow cytometric analysis of CD45^+^ T cells (a), CD4^+^T cells (b), CD8^+^ T cells (c), MDSCs (d), and M1 cells and M2 cells (e) in tumors. CTRL: control; RT: radiotherapy; IO: immunotherapy; AN: anlotinib.

**Figure 4:**
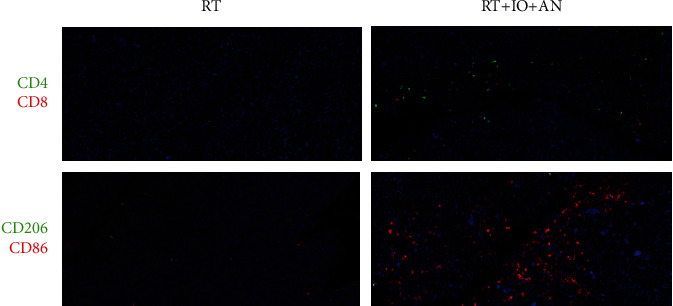
Triple therapy (radiotherapy combined with anti-PD-L1 and anlotinib) reversed the immunosuppressive effect of radiation on the tumor microenvironment. CTRL: control; RT: radiotherapy; IO: immunotherapy; AN: anlotinib.

RT was reported to alter the tumor immune microenvironment remarkably. We first analyzed the different immune cells, including CD4^+^ T cells (CD45^+^, CD3^+^, and CD4^+^), CD8^+^ T cells (CD45^+^, CD3^+^, and CD8^+^), myeloid-derived suppressor cells (MDSCs) (CD45^+^, CD11b^+^, and Gr-1^+^), M1 type tumor-associated macrophages (M1 cells) (CD45^+^, CD11b^+^, F4/80^+^, and CD86^+^), and M2 type tumor-associated macrophages (M2 cells) (CD45^+^, CD11b^+^, F4/80^+^, and CD206^+^) in the tumor immune microenvironment of control and RT groups by flow cytometry. Analysis showed that radiation augmented the total immune cell infiltration (CD45^+^ cells: 5.6 ± 0.2 vs. 27.1 ± 0.4, *p* < 0.0001). In terms of the subsets, we found that CD4^+^ T cells, CD8^+^ T cells, and M1 cells significantly decreased following RT (CD4^+^ T cells: 17.7 ± 0.2 vs. 7.4 ± 0.1, *p* < 0.0001; CD8^+^ T cells: 6.0 ± 0.3 vs. 3.6 ± 0.1, *p* = 0.0012; M1 cells: 4.0 ± 0.6 vs. 1.71 ± 0.1, *p* = 0.0258) (Figure 3). On the other hand, the percentage of MDSCs and M2 cells in the tumor tissue significantly increased after RT (MDSCs: 15.1 ± 2.8 vs. 26.4 ± 0.7, *p* = 0.0194; M2 cells: 28.0 ± 2.8 vs. 45.2 ± 0.4, *p* = 0.0034) (Figure 3). These data indicate that radiation changed the immune cell distribution in the tumor environment, that is, the infiltration of positive immune cells such as CD8^+^ T cells and M1 cells reduced while the suppressor cells such as MDSCs and M2 cells increased. And this can also be demonstrated in the IF (Figure 4).

### 3.3. Trinity Strategy Could Significantly Potentiate the Tumor Immune Microenvironment Compared with Other Treatment Combinations

To explore which strategy has the potential to attenuate and reverse the suppressive immune microenvironment, we treated the mice with RT, anti-PD-L1, RT+anlotinib, RT+anti-PD-L1, or RT+anti-PD-L1+anlotinib. The results of flow cytometry illustrated that the trinity strategy brought about the most positive tumor immune microenvironment. All treatments could enhance the total immune cell infiltration comparing with control group. Both RT+anlotinib and RT+anti-PD-L1+anlotinib could significantly increase the CD4^+^ T cells comparing with control (CD4^+^ T cells: 37.6% ± 5.9% vs. 17.7% ± 0.2%, *p* = 0.01; 31.9% ± 2.1% vs. 17.7% ± 0.2%, *p* = 0.0005), whereas RT, anti-PD-L1, and RT+anti-PD-L1 decreased the CD4^+^ T cells in tumor microenvironment. The trinity strategy significantly increased CD8^+^ T cell infiltration (*p* = 0.004), with the percentage of 14.1% that was more than any other treatment. The percentage of M1 cells in the triple therapy was also significantly higher (M1 cells: 38.2% ± 7.2% vs. 4.0% ± 0.6%, *p* = 0.0024) and was the most among all treatment groups. MDSCs significantly decreased in the triple therapy compared with RT (MDSCs: 15.2% ± 6.1% vs. 26.4% ± 1.3%, *p* = 0.0358). The percentage of M2 cells in the triple therapy group was remarkably reduced comparing with RT (M2 cells: 4.9% ± 2.2% vs. 45.2% ± 0.4%, *p* < 0.0001) and was the lowest amongst all treatments (Figure 3). These can also be found in the IF (Figure 4). In brief, triple therapy significantly promoted the filtration of CD45^+^, CD8^+^ T cells, and M1 cells and decreased MDSCs and M2 cells. To conclude, the trinity strategy of combining RT, anti-PD-L1, and anlotinib not only has the best antitumor activity but also can reverse the suppressive immune microenvironment induced by radiotherapy and significantly potentiate the tumor immune microenvironment compared with all the other treatments.

### 3.4. Trinity Strategy Promoted Function of Immune Cells and the Positive Immune Environment in LLC Tumors

**Figure 5:**
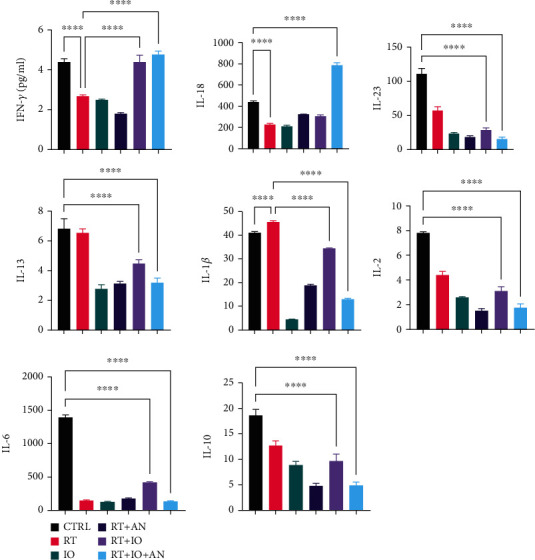
Levels of cell cytokines associated with the activity and function of immune cells. CTRL: control; RT: radiotherapy; IO: immunotherapy; AN: anlotinib.

**Figure 6:**
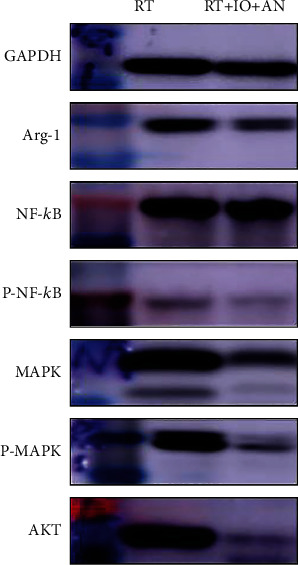
Expressions of Arg-1, NF-*κ*B p65, p-NF-*κ*B p65, MAPK, p-MAPK, and AKT in subcutaneous tumors from mice treated with radioimmunotherapy or triple therapy. RT: radiotherapy; IO: immunotherapy; AN: anlotinib.

To identify the activity and function of immune cells, we performed quantitative detection of cell cytokines. The levels of IFN-gamma and IL-18 were the highest in the triple therapy group, while the level of IL-23 was the lowest in the triple therapy group (Figure 5). Besides, IL-13, IL-1 beta, IL-2, IL-6, and IL-10 were significantly reduced in the triple therapy group (Figure 5). We also conducted the Western blot to examine the level of Arg-1 in the mice tumor tissues. We found that the trinity strategy led to a marked decrease in Arg-1 compared with radioimmunotherapy (Figure 6).

### 3.5. Triple Therapy Promoted the Antitumor Effect of Radioimmunotherapy via Downregulation of NF-*κ*B, MAPK, and AKT Pathways

We also explored the mechanism underlying the synergistic effect of anlotinib and radioimmunotherapy. Previous studies have demonstrated that nuclear factor kappa B (NF­*-κ*B) functions in many cellular constituents of the tumor microenvironment and modulates inflammation, tumorigenesis, and metastasis; therefore, we first hypothesized that triple therapy might play a role in blocking NF­*-κ*B activation. Analysis of levels of NF­*-κ*B p65 and p-NF-­*κ*B p65 in LLC subcutaneous tumors from mice treated with radioimmunotherapy or triple therapy revealed that the triple therapy decreased levels of p-NF­*-κ*B, suggesting the addition of anlotinib blocking activation of the NF­*-κ*B pathway (Figure 6). Mitogen-activated protein kinase (MAPK) and AKT pathways have also been identified as pivotal regulators of tumor immune microenvironment. We second tested these two signaling pathways. Results of Western blot showed that both of them were decreased in the triple therapy (Figure 6).

## 4. Discussion

The combination of radiotherapy and immunotherapy has become one of the most promising strategies for cancer treatment. How to optimize it is currently a hotspot, including how to further improve the response rate, scale up the potential population who can benefit and reduce the incidence rate of adverse events related to combination treatment. Antiangiogenic therapy is an important component of targeted therapy, and in combination treatment, it may further prolong survival and improve the quality of life [13]. The present study is aimed at confirming the positive role of antiangiogenic therapy on the combination of radiotherapy and immune checkpoint inhibitors in Lewis lung carcinoma mouse and extending understanding of the potential mechanisms from aspects of tumor immune microenvironment.

Our study demonstrated that anlotinib enhanced the therapeutic effect of radioimmunotherapy, and the triple therapy achieved the best antitumor activity compared with other combination treatments. We found that trinity strategy could significantly potentiate the tumor immune microenvironment compared with other treatment combinations. In addition, triple therapy might promote the antitumor effect of radioimmunotherapy via downregulation of MAPK, AKT, and NF-*κ*B pathways.

The combination of radiotherapy and immunotherapy has been proved in numerous preclinical and clinical studies to have obvious synergistic effects. Immune checkpoint inhibitor (ICI) can enhance the antitumor immunity caused by radiotherapy: due to the immunosuppressive microenvironment already existing in the tumor, radiotherapy alone cannot produce sufficient antitumor immune response. Therefore, the abscopal effect is rare to occur clinically when radiotherapy is implemented alone. CTLA-4 or PD-1/PD-L1 monoclonal antibody can enhance the radiotherapy-induced in situ vaccination [18, 19]. On the other hand, radiotherapy increases the effect of ICI: although CTLA-4 and PD-1/PD-L monoclonal antibody can reverse the inhibition of T cells in the immunosuppressive tumor microenvironment to some extent, the activation of T cells still depends on antigen stimulation and participation of activated costimulatory molecule CD28 on the surface of mature APC. Radiotherapy can increase the expression and presentation of tumor antigen and consequently amplify the immune response induced by ICI. Radiotherapy can also improve the immunomodulatory effect by increasing CTL infiltration, enhancing CTL activity, enlarging antigen peptide pool, and triggering the diffusion of tumor antigen determinants [20]. For unresectable stage III NSCLC, concurrent chemoradiotherapy is the basis of standard treatment. In order to improve the efficacy of chemoradiotherapy, many attempts, including increasing induction chemotherapy, consolidation chemotherapy, and radiotherapy dose, all ended in failure. The Pacific study confirmed that the consolidation of anti-PD-L1 inhibitor durvalumab on the basis of chemoradiotherapy can further improve the overall survival and progression-free survival [12]. Based on this, the treatment regimen was written into the NCCN guidelines as class I recommendation in 2018. Even though immunotherapy combined with radiotherapy displayed a promising antitumor treatment benefit on NSCLC, preclinical or clinical studies to investigate the efficacy of trinity strategy of combining antiangiogentic therapy with radioimmunotherapy are still rare, and the results are immature. Especially in hypofractionated radiotherapy, the reoxygenation utilization rate is lower than that of conventional fractionated radiotherapy. Low fractionated and high-dose radiotherapy lead to an increase in the ratio of hypoxic cells and high expression of VEGF. It can be inferred that the sensitization effect of anlotinib might be more significant in hypofractionated radiotherapy [21].

We innovatively added anlotinib to radioimmunotherapy to investigate whether the trinity strategy can exhibit even superior efficacy. The triple therapy showed the most obvious antitumor effect in all treatments. Then, we detected the tumor immune phenotype of different treatments and found that the trinity strategy led to the most positive immune phenotype in tumor tissue compared with other treatment combinations, which is consistent with the efficacy. The addition of anlotinib to radioimmunotherapy boosted the infiltration of CD8^+^ T cells in mice tumor tissues, and the amount of M1 cells also peaked in the triple therapy group, which is significant to the antitumor immune responses. On the other hand, the triple therapy obviously reduced the number of immunosuppressive cells. We found that the infiltration of MDSCs decreased in the triple therapy compared with radiotherapy and other radiotherapy combination treatments. The level of M2 cells in tumor tissues was the lowest in triple therapy.

We next determined the levels of cell cytokines associated with the activity and function of immune cells. The levels of IFN-gamma was the highest in the triple therapy group, which was secreted primarily by activated lymphocytes such as CD4^+^ and CD8^+^ T cells [22], gamma delta T cell [23], as well as NK[24] and NK T cells [25]. Numerous antitumor effects of IFN-*gamma* have been described, including regulation of antigen presentation, promotion of inflammatory and chemotactic signals, activation and polarization of responding leukocytes, recruiting effector leukocytes, as well as direct antiproliferative and antiangiogenic effects [26]. The level of IL-23 was showed to be the lowest in the triple therapy group, which was mainly secreted by myeloid cells and associated with suppressive immune microenvironment, tumor cell proliferation, and treatment resistant [27]. Besides, IL-13, IL-1 beta, IL-2, IL-6, and IL-10 were significantly reduced in the triple therapy group (Figure 5). Local signals IL-13, IL-6, and IL-10 in the tumor microenvironment were reported to polarize macrophages into protumoral M2-like cells [28], which was in line with the results of flow cytometry. Tumor-derived IL-1 has been proven to have a role in modulating the composition of TME by recruiting MDSCs, TAM, TAN, Breg cells, and Th17, contributing to the angiogenic switch sustaining the production of angiogenic factors such as VEGF [29]. IL-1 has also been emerging as a cytokine involved in drug resistance [30]. We also conducted the Western blot to examine the level of Arg-1 in mice tumor tissues and found that Arg-1 was substantially decreased in the triple therapy compared with radioimmunotherapy. Elevated levels of Arg-1 expressed by MDSC were reported to mediate the depletion of L–arginine from the TME that leads to a cell cycle arrest in T cells and T cell anergy due to the downregulation of TCR zeta-chain expression [31].

NF-*κ*B, MAPK, and AKT are main signal pathways associated with tumor immune microenvironment. Activating of these pathways was demonstrated to induce an immune-tolerant tumor microenvironment [28, 30, 32]. We found that the expression levels of p-NF-*κ*B, MAPK, p-MAPK, and AKT were reduced in triple therapy compared with radioimmunotherapy, which indicated that the best antitumor effect and the most positive immune microenvironment of trinity strategy might be associated with downregulation of NF-*κ*B, MAPK, and AKT pathways.

There are some limits in our study. First, the influence on survival and tumor recurrence were not observed; second, only one cell line was used in the study; third, whether changing the radiation dose-fractionation will affect the efficacy of triple therapy has not been discussed. Despite these limitations, taken all these benefits of anlotinib combined with radioimmunotherapy together, our data implicated that antiangiogentic therapy could be a promising synergetic treatment for radioimmunotherapy in patients with NSCLC. Although our study was of high clinical significance, the combination of antiangiogentic therapy with radioimmunotherapy merits evaluation on clinical trials.

## 5. Conclusion

In summary, we have demonstrated that the addition of anlotinib to radioimmunotherapy increased the infiltration of CD8^+^ T and M1 cells and reduced MDSCs and M2 cells in LLC tumors. Moreover, the combination of anlotinib with radioimmunotherapy led to an increase in the levels of IFN-gamma and IL-18 and descending in IL-23, IL-13, IL-1 beta, IL-2, IL-6, IL-10, and Arg-1. Thus, anlotinib significantly improved the tumor immune microenvironment combined with radioimmunotherapy. As a consequence, compared with radioimmunotherapy, anlotinib plus radioimmunotherapy displayed better therapeutic efficacy in tumor control in tumor-bearing mice.

Our findings have a high clinical significance. Based on preliminary translational observations, our work raised the possibility that antiangiogentic therapy might be a potential synergistic modality for radioimmunotherapy in NSCLC patients. Remarkably, this study provides a basis for future clinical studies into the treatment of NSCLC.

## Data Availability

The data used to support the findings of this study are available from the corresponding author upon request.
